# Giant Enhancement of the Anomalous Transverse Magneto‐Optical Kerr Effect in a Magneto‐Optical Grating

**DOI:** 10.1002/nap2.70139

**Published:** 2026-05-14

**Authors:** Zicheng Yang, Shuqiao Xu, Haoshuang Zhong, Guoyong Zhang, Hao Chen, Peng Zhan, Shuming Wang, Zhuo Chen, Zhenlin Wang

**Affiliations:** ^1^ School of Physics and National Laboratory of Solid State Microstructures Nanjing University Nanjing China

**Keywords:** guided mode resonance, high‐Q, quasi‐bound states in the continuum, the anomalous transverse magneto‐optical Kerr effect

## Abstract

The anomalous transverse magneto‐optical Kerr effect (TMOKE) under s‐polarization is typically suppressed by symmetry constraints, leading to negligible signals. Here, we propose an indirect magneto‐optical (MO) coupling mechanism that originates from the spatially asymmetric near‐field distribution of resonant modes, rather than from the direct coupling between the incident field and the MO tensor. Guided by this principle, we design a quasi‐two‐dimensional MO grating that strategically coordinates resonances along two orthogonal directions. Specifically, modes perpendicular to the grating ridges establish a wide‐angle broadband near‐zero reflection background to satisfy the rigorous mathematical condition for maximum TMOKE, whereas the orthogonal high‐Q resonances are responsible for inducing maximal near‐field asymmetry. This allows us to push the s‐polarized TMOKE to approach its theoretical limit (∼2)—a performance leap of over an order of magnitude compared to previous reports. The proposed structure exhibits exceptional robustness against material losses and fabrication tolerances, alongside a broad angular tolerance (up to 40°), offering a practical blueprint for high‐performance nonreciprocal photonic devices.

## Graphical abstract

We demonstrate an indirect magneto‐optical coupling mechanism that overrides the symmetry‐induced suppression of s‐polarized TMOKE. A quasi‐two‐dimensional grating coordinates orthogonal resonances to establish a near‐zero reflection background and maximal near‐field asymmetry, achieving a theoretical‐limit TMOKE of ∼2. This represents a giant improvement over previous reports while offering excellent robustness against material losses, fabrication tolerances, and wide incident angles.

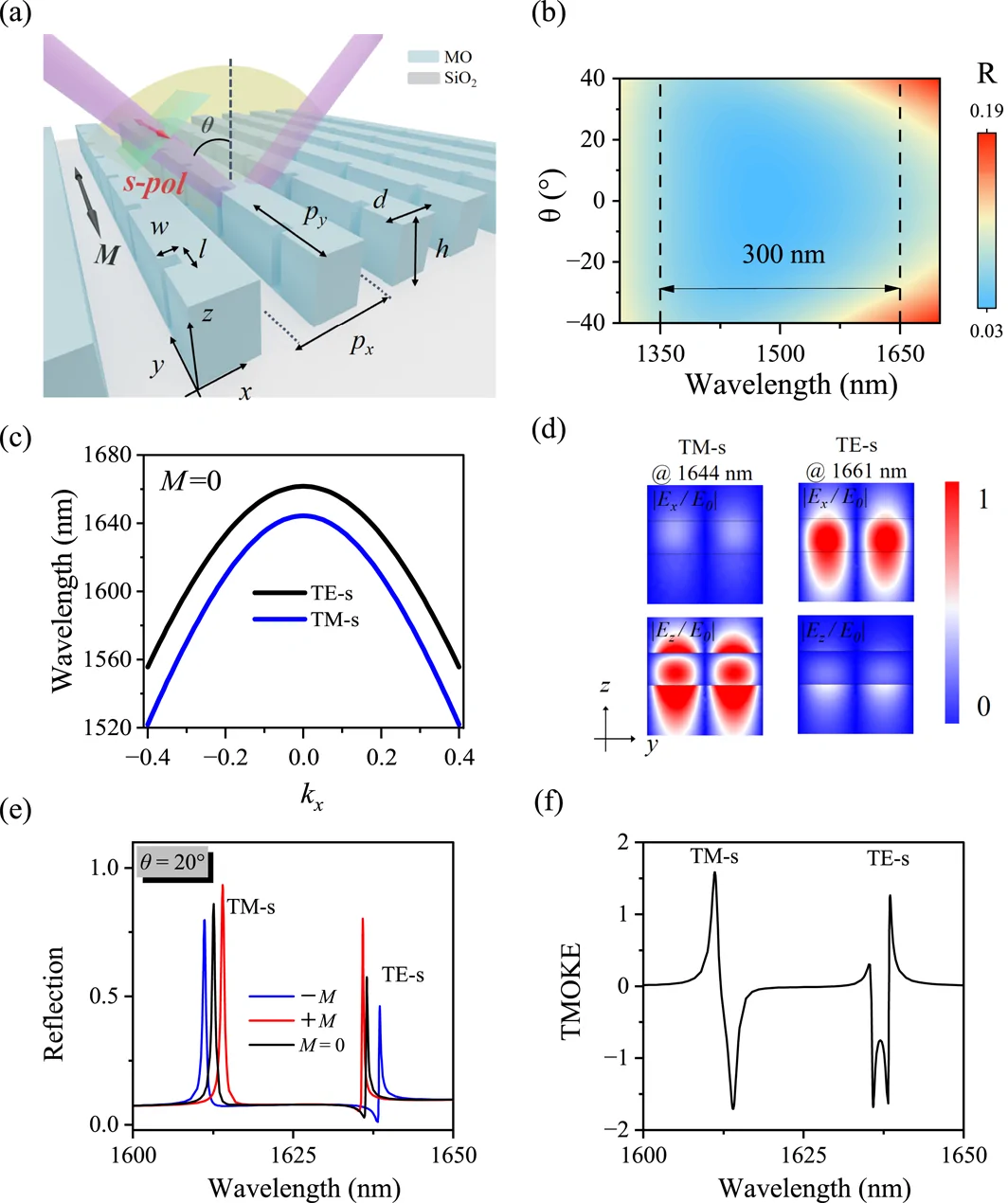

## 1 Introduction

Magneto‐optical (MO) effects serve as a fundamental physical bridge between optics and magnetism, providing the physical cornerstone for high‐sensitivity magnetic sensing and nonreciprocal photonic devices [1, 2, 3, 4, 5, 6, 7, 8, 9, 10, 11]. Among these, the transverse magneto‐optical Kerr effect (TMOKE) has garnered significant interest due to its unique ability to modulate reflectance without altering the polarization state of light [12]. TMOKE is quantitatively characterized by the normalized reflectance difference under reversed magnetization directions: *TMOKE = 2(R*_*−M*_ *− R*_*+M*_ *)/(R*_*+M*_ *+ R*_*−M*_) [13].

TMOKE signals in conventional MO materials are intrinsically weak (∼10^−3^–10^−5^) due to the small off‐diagonal components of the permittivity tensor [14]. This inherent limitation severely restricts the signal‐to‐noise ratio and detection sensitivity in practical applications, especially for wide‐angle and multichannel modulation [14, 15]. Therefore, over the past few years, many attempts have been made and various resonant modes—including surface plasmon resonances (SPRs) [16, 17, 18, 19], Mie resonances [20], waveguide modes [15], optical Tamm modes [21], Fabry–Perot modes [22], and bound states in the continuum (BICs) [23]—have been exploited to enhance the TMOKE response.

However, most existing literature has predominantly focused on the enhancement of p‐polarized TMOKE, primarily because the incident electric field of p‐polarized light contains both in‐plane and out‐of‐plane components that can directly couple to the off‐diagonal MO tensor elements. In these p‐polarized systems, the enhanced local field interacts directly with the MO coupling channels, allowing the TMOKE response to reach high levels and even approach its theoretical limit in specific nanostructures [24]. In sharp contrast, the incident electric field under s‐polarization is purely transversal, which is inherently decoupled from the MO‐active transitions in traditional frameworks. Consequently, s‐polarized TMOKE is typically considered symmetry‐forbidden or negligible, and achieving giant s‐polarized TMOKE remains a more fundamental and formidable challenge. Even if strong resonant modes are supported, the s‐polarized TMOKE signal typically remains suppressed unless the local field distribution effectively breaks the spatial symmetry within the MO layer. Although several recent studies have explored s‐polarized TMOKE using iron‐garnet nanopillars [25], cobalt nanostructures [26], or metallic gratings [27], the reported enhancement levels remain far below 1.

In this work, we propose a mode‐driven “indirect magneto‐optical coupling” mechanism to overcome these limitations. In this paradigm, s‐polarized TMOKE originates from the spatially asymmetric near‐field distribution of resonant modes within the MO layer, rather than from the direct coupling of the incident field to the permittivity tensor. To manifest this mechanism and reach near‐limit performance, we specifically design a quasi‐two‐dimensional (quasi‐2D) MO grating—comprising a 1D grating with periodic air grooves etched into the ridges—to support spatially orthogonal resonances. Our design follows a functional decoupling strategy: first, we utilize guided‐mode resonances (GMRs) perpendicular to the grating ridges to establish a wide‐angle, near‐zero reflection “dark background,” thereby satisfying the rigorous mathematical condition for maximum TMOKE. Second, the introduction of periodic grooves along the ridges enables the excitation of high‐Q quasi‐BICs and GMRs that induce maximal near‐field asymmetry within the MO layer. This synergistic coordination allows us to push the anomalous s‐polarized TMOKE to approach its theoretical limit (∼2). Furthermore, the proposed structure operates effectively for p‐polarized light, achieving a similarly near‐limit TMOKE response. Combined with its exceptional robustness against material losses and fabrication deviations, as well as a broad angular tolerance (up to 40°), our design offers a practical blueprint for high‐performance polarization‐insensitive nonreciprocal photonic devices.

## 2 Materials and Methods

To identify the optimal structural configurations and ensure numerical rigor, we developed a collaborative framework that combines rigorous coupled‐wave analysis (RCWA) with the finite‐element method (FEM). The RCWA method, integrated with particle swarm optimization (PSO), was specifically employed to optimize the 1D dielectric grating. This RCWA–PSO framework is designed for high‐efficiency parameter sweeps across a multi‐dimensional space (including grating period, ridge width, and depth). The optimization target was to minimize broadband reflectance, establishing the critical near‐zero reflection background required for both polarizations across a wide angular range.

The FEM method (COMSOL Multiphysics) was used to model the quasi‐2D structure, perform eigenmode analysis, and generate high‐resolution spatial field distributions. In the simulations, periodic boundary conditions (PBCs) were applied in the *x*‐and *y*‐directions. To absorb reflected and transmitted light without parasitic reflections, one‐wavelength‐thick perfectly matched layers (PMLs) were placed at the top and bottom of the calculation domain. To ensure convergence and reproducibility, an extremely fine mesh was adopted, particularly near the grooves, where the maximum element size was kept below one‐fifth of the smallest structural feature.

## 3 Result

Figure 1a illustrates the proposed quasi‐2D MO grating designed for efficient TMOKE enhancement. The design begins with a foundational 1D bismuth iron garnet (BIG, *ε* = 6.25) MO grating on a SiO_2_ substrate (*n* = 1.45), characterized by a period *p*_*x*_ = 445 nm, a ridge height *h* = 326 nm, and a ridge width *d* = 356 nm. To transform this into a quasi‐2D system, periodic air grooves (length *l* = 100 nm, width *w* = 100 nm) are subsequently etched into each grating ridge along the *y*‐direction, with a period *p*_*y*_ = 1050 nm. Figure 1b displays the reflection map for the foundational 1D MO grating under s‐polarized incidence. The results reveal that this optimized 1D structure provides a broadband and wide‐angle low‐reflectance region, where the reflectance is below 10% across a 300 nm bandwidth (from 1350 to 1650 nm) for incidence angles ranging from −40° to +40°. This robust low‐reflection environment serves as an optimized near‐zero reflection background for the subsequent resonance excitation along the ridges (*y*‐direction), acting as a critical prerequisite for pushing the TMOKE response toward its theoretical limit across a broad angular and spectral range.

**FIGURE 1:**
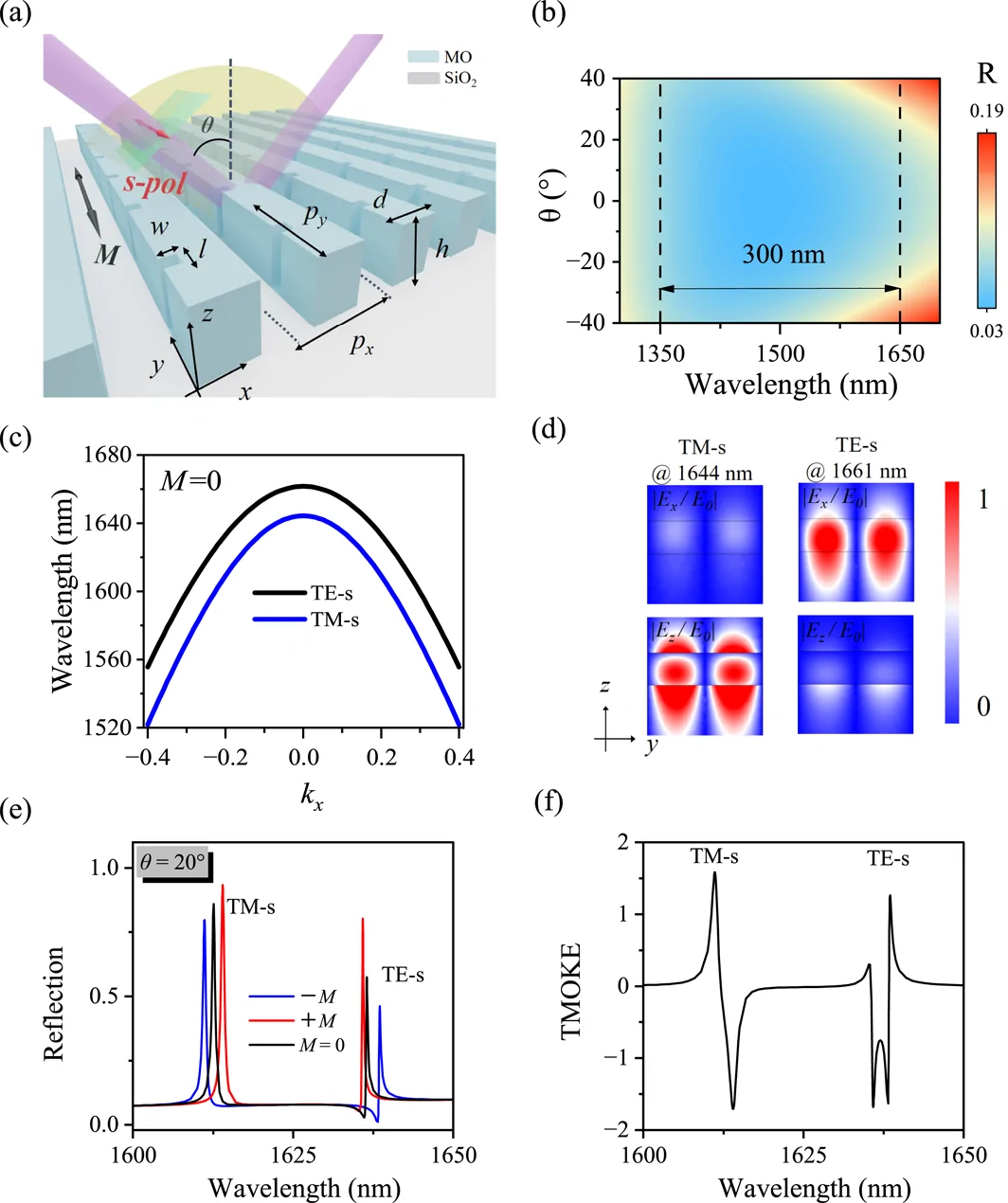
Schematic illustration of the quasi‐2D MO grating and its giant TMOKE response. (a) Schematic of the quasi‐2D MO grating with periodic air grooves etched into the grating ridges, situated on a SiO_2_ substrate. (b) Reflection map of the foundational 1D MO grating under s‐polarized incidence as a function of wavelength and incidence angle. (c) Dispersion relations of the resonant leaky modes in the quasi‐2D MO grating along the *k*_*x*_ direction. (d) Normalized electric field magnitudes of the TE‐s and TM‐s modes at the *yoz* cross‐section (*x* = *p*_*x*_/2). (e) Reflection spectra under reversed magnetization states (± *M*) and zero field (*M* = 0) at *θ* = 20°. (f) Corresponding TMOKE spectrum for s‐polarization at *θ* = 20°.

The introduction of periodic grooves provides the necessary reciprocal lattice vectors to compensate for the momentum mismatch between free‐space photons and the guided modes of the MO layer along the *y*‐direction [28, 29]. Consequently, the originally bound guided modes are converted into radiative leaky modes, manifested as sharp GMRs or quasi‐BICs in the far field [30]. Figure 1c presents the band structure of the quasi‐2D MO grating calculated using the FEM method. For clarity, only the TE‐like and TM‐like mode branches that are active under s‐polarized excitation are presented, labeled as TE‐s and TM‐s, respectively. Both branches exhibit a characteristic blue shift as the incidence angle increases, demonstrating the continuous spectral tunability of these resonances. Figure 1d displays the normalized electric field magnitudes (*|E*_*x*_ */E*_*0*_ *|* and *|E*_*z*_ */E*_*0*_ *|*) for the TE‐s and TM‐s modes at the *Γ* point. The field profiles are captured at the *yoz* cross‐section taken at the center of the unit cell along the *x*‐axis. As expected, the TE‐s and TM‐s modes are dominated by *E*_*x*_ and *E*_*z*_ components, respectively.

To investigate the TMOKE response of the quasi‐2D MO grating, the system is configured in the Voigt geometry, with an external magnetic field applied along the *y*‐axis, perpendicular to the plane of incidence. Under a *y*‐oriented external magnetic field, the permittivity of the MO material changes from a scalar to a tensor with nonzero off‐diagonal elements.

$$\varepsilon \left(r\right)=\left(\begin{array}{ccc} \varepsilon & 0 & -ig\\ 0 & \varepsilon & 0\\ ig & 0 & \varepsilon \end{array}\right)$$

To simplify the model, the tensor components *ε* and *g* are assumed to be constant within the operating spectral range, where *ε* = 6.25 (consistent with the 1D grating) and *g* = 0.06. This *g*‐value is consistent with experimentally reported off‐diagonal elements for BIG under magnetic saturation (∼0.06 T) [31]. Although initial calculations assume a lossless model to define the ideal theoretical limit, the impact of intrinsic losses is thoroughly validated later to ensure experimental relevance.

Under s‐polarized incidence at *θ* = 20°, Figure 1e displays the reflection spectra under reversed magnetization (± *M*). For direct comparison, the reflection spectrum in the absence of an external magnetic field (*M* = 0) is also presented. The introduction of off‐diagonal elements by the external magnetic field alters the electromagnetic boundary conditions and modulates the resonance condition of the modes, resulting in opposite frequency shifts upon magnetization reversal. As shown in Figure 1e, the reflection peak of the TM‐s mode blueshifts from *λ* = 1612.5 nm (*M* = 0) to *λ* = 1611.1 nm under +M, whereas its redshifts to *λ* = 1614 nm under −*M*. Similarly, the reflection peak of the TE‐s mode redshifts from *λ* = 1636.5 nm (*M* = 0) to *λ* = 1638.5 nm under +*M*, but blueshifts to *λ* = 1635.8 nm under −*M*. Although the relative spectra shift between two magnetization states is typically small (∼2.9 nm for TM‐s and ∼2.7 nm for TE‐s), this shift is pronounced and fully resolved in our structure due to the high‐quality factors (Q ∼ 10^3^) of the resonant modes. Specifically, the linewidths of the TE‐s and TM‐s modes are 0.4 and 0.7 nm, respectively, which are significantly narrower than the magnitude of the MO‐induced frequency shift. This ensures that the two magnetization states are effectively decoupled in the spectral domain, allowing the reflectance to “toggle” between a resonant peak and the near‐zero reflection background upon magnetization reversal.

Crucially, the synergy between this spectral shift and the established 1D low‐reflection background allows one of the reflection states (e.g., under −*M* for the TM‐s mode) to reach a near‐zero minimum, satisfying the rigorous condition (*R* ≈ 0) for maximum TMOKE. As a result, the TMOKE spectrum in Figure 1f reaches a giant peak value approaching the theoretical limit of 2. Around 1612 nm, the S‐shaped line shape TMOKE originates from the magnetization‐induced spectral shift of the TM‐s resonance. The small magneto‐optical shift is converted into reflection differences with opposite signs. On one side of the resonance, R (−*M*) is larger than R (+*M*), giving a positive TMOKE response. On the other side, R (−*M*) becomes smaller than R (+*M*), leading to a negative TMOKE response. Therefore, the TMOKE signal shows a clear sign reversal near the resonance and forms a typical S‐shaped line shape. Near 1635 nm, the TMOKE spectrum shows a saddle‐like line shape. This feature mainly arises from the Fano‐type resonance. Because the Fano resonance has an asymmetric peak‐dip profile, a small magneto‐optical shift is converted into alternating positive and negative reflection differences. The resulting TMOKE reaches about 1.7, close to the theoretical limit of 2, which represents a performance leap of over an order of magnitude compared to previous reports. The low‐reflectance grating we designed exhibits significant performance advantages. A comparison with a regular grating is provided in the Supporting Information S2.

The realization of an anomalous s‐polarized TMOKE response approaching its theoretical limit prompts a fundamental question regarding its underlying physical origin. As established earlier, s‐polarized incident light is inherently decoupled from the off‐diagonal elements of the MO permittivity tensor in conventional frameworks. To resolve this apparent contradiction, we shift our focus from far‐field performance to the localized field‐matter interactions, specifically investigating how oblique incidence acts as a trigger to induce the asymmetric resonant near‐fields required for the indirect MO coupling.

Figure 2a displays the electric field components *E*_*x*_ */E*_*0*_ and *E*_*z*_ */E*_*0*_ of the TM‐s mode under resonance at *λ* = 1612.5 nm and *θ* = 20°. Given that the mode propagates along the grating ridges (*y*‐direction), the field distributions maintain mirror symmetry relative to the *x*‐axis; however, oblique incidence introduces a pronounced spatial imbalance along the *x*‐direction. The resonant fields within the MO layer exhibit a significant intensity shift toward one side of the unit cell. To quantitatively characterize this spatial imbalance, we introduce a field asymmetry factor (*β*). Recognizing that different electric field components contribute differently to the MO coupling, we calculate *β* for the *E*_*x*_ and *E*_*z*_ components separately. This factor is obtained by integrating the field intensity over the left and right halves of the MO layer relative to the *yoz* symmetry plane.

$$\beta_{j}=\frac{\left|\int_{V_{\text{left}}}\frac{E_{j}\left(r\right)}{E_{0}}dV-\int_{V_{\text{right}}}\frac{E_{j}\left(r\right)}{E_{0}}dV\right|}{V_{\text{total}}}$$

where *j* = *x* or *z*, and *V*_left_ and *V*_right_ denote the left and right regions of the MO layer within a single unit cell, respectively. *V*_total_ represents the total volume of the MO layer within the unit cell (*V*_total_ = *V*_left_ + *V*_right_), serving as a volume normalization factor to quantify the volume‐averaged asymmetric field intensity.

**FIGURE 2:**
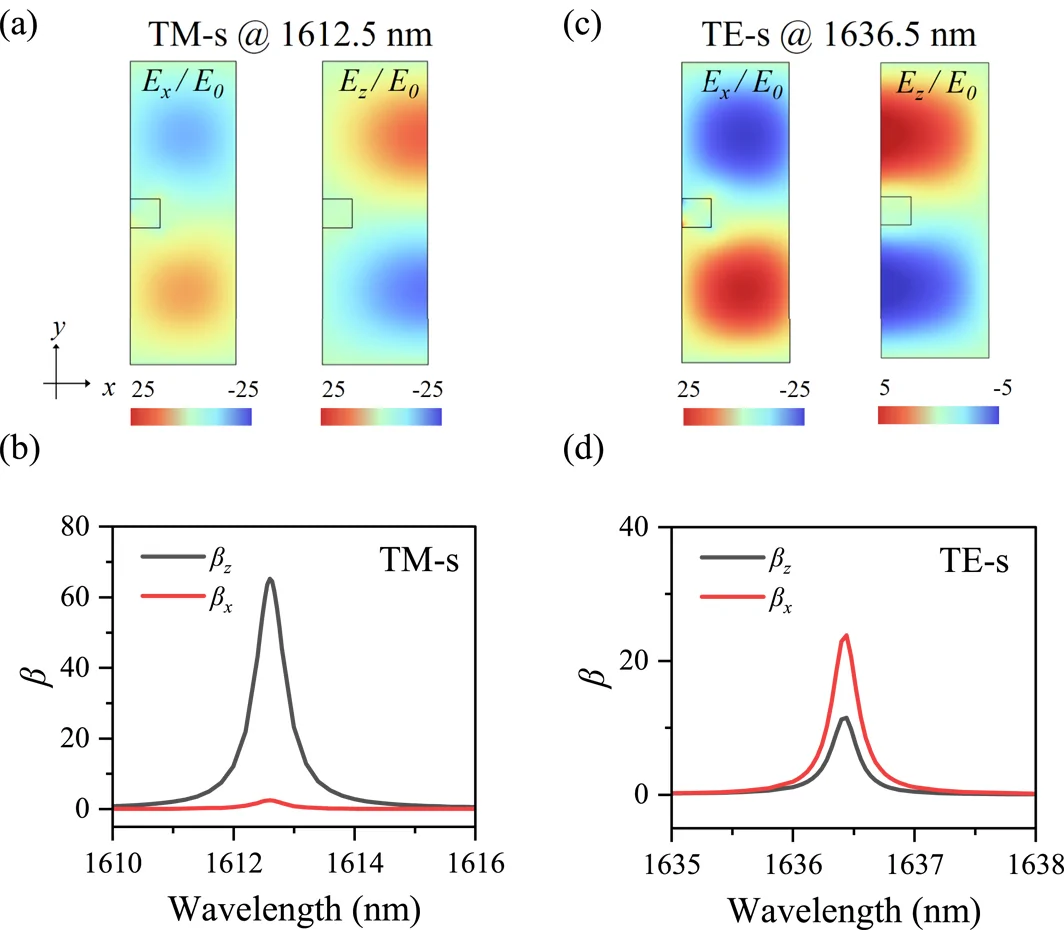
Electric field distributions and asymmetry factors of the TM‐s and TE‐s modes. Normalized electric field components of the TM‐s (a) and TE‐s (c) modes at the resonant wavelengths. Wavelength dependent asymmetry factor of the electric field components in the TM‐s (b) and TE‐s (d) modes.

The calculated asymmetry spectra for the TM‐s mode are presented in Figure 2b. The results reveal that the out‐of‐plane component *E*_*z*_ is the dominant constituent and exhibits the highest degree of asymmetry, with its *β*_*z*_ value reaching a peak of approximately 65 at the resonance wavelength. In contrast, the component *E*_*x*_ remains relatively weak with negligible asymmetry. This confirms that the TM‐s mode effectively concentrates a highly imbalanced *E*_*z*_ field within the MO layer, preventing the spatial cancellation of magneto‐optical contributions and thereby providing the physical foundation for the giant TMOKE response.

A similar analysis was performed for the TE‐s mode, as shown in Figure 2c,d. For this mode, the dominant field constituent shifts to the in‐plane *E*_*x*_ component, which exhibits a peak *β*_*x*_ value of approximately 25 at resonance, accompanied by moderate asymmetry in *E*_*z*_ (*β*_*z*_ ≈ 10). Although the dominant components and their respective asymmetry strengths differ, both modes consistently support the asymmetric local field distributions required for efficient indirect coupling. The quantitative correlation between these peak asymmetry factors and the observed TMOKE maxima confirms that this mode‐induced near‐field asymmetry is the universal driver for the anomalous TMOKE enhancement in our quasi‐2D system.

To evaluate the versatility and robustness of the proposed mechanism, we investigate the s‐polarized TMOKE response under varying structural and excitation parameters. Figure 3a–c presents the s‐polarized reflectance spectra under reversed magnetization (± *M*) at *θ* = 20° for different longitudinal periods, *p*_*y*_. As *p*_*y*_ increases from 900 to 1000 nm, the reflection peaks exhibit a clear redshift, consistent with the waveguide dispersion relation. The absolute TMOKE values as a function of *p*_*y*_ are summarized in Figure 3d, where four distinct clusters of data points represent the TMOKE values calculated at four reflection peaks. Specifically, the black squares and red circles represent the absolute TMOKE values at the R (−*M*) and R (+*M*) peaks of the TM‐s mode, respectively. Blue and green triangles represent the corresponding values at the R (+*M*) and R (−*M*) peaks of the TE‐s mode, respectively. Notably, although the TE‐s mode is weakly excited under certain parameters, the TM‐s mode still yields high TMOKE values across this range. This demonstrates that the resonance position can be continuously tuned by the grating period while maintaining near‐limit performance.

**FIGURE 3:**
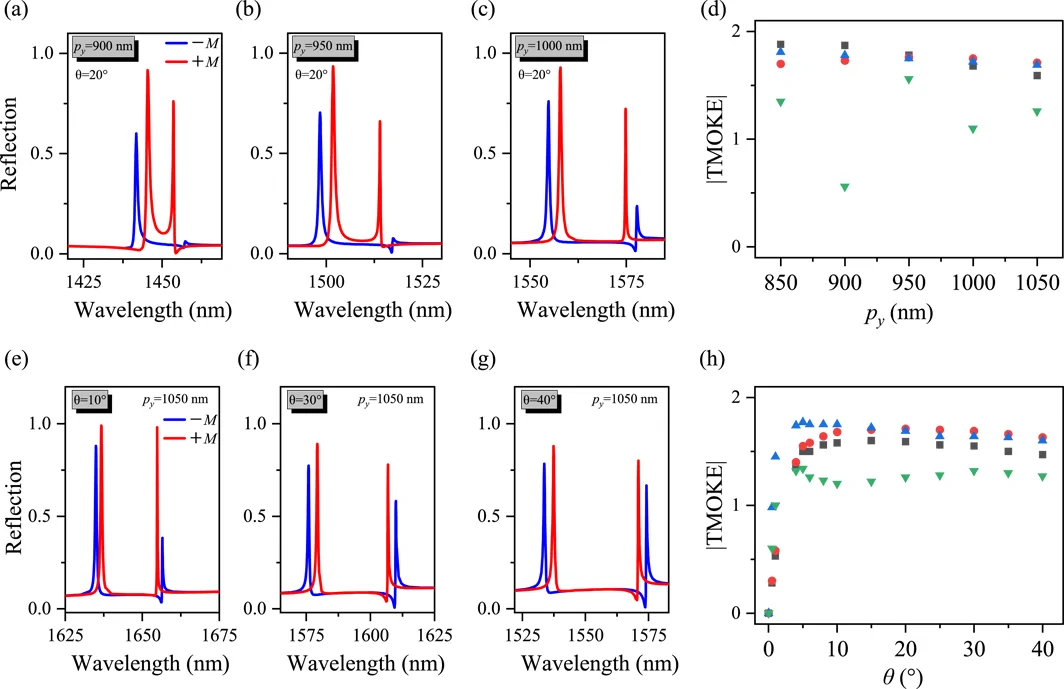
Reflection and TMOKE spectra under s‐polarized incidence. (a)–(c) Simulated reflection spectra of the magneto‐optical grating and (d) |TMOKE| obtained at different *p*_*y*_. (e)–(g) Simulated reflection spectra of the magneto‐optical grating and (h) |TMOKE| obtained at different incident angles.

Figure 3e–g shows the reflection spectra at *p*_*y*_ = 1050 nm for varying incidence angles. All resonances exhibit a systematic blue shift as *θ* increases from 10° to 40°. According to the definition *TMOKE = 2(R*_*−M*_ *− R*_*+M*_ *)/(R*_*+M*_ *+ R*_*−M*_), the giant response in our structure stems from the fully resolved spectral splitting on a near‐zero reflection background. Unlike conventional structures where *ΔR* is typically on the order of 0.01, our synergistic design ensures that the reflectance “toggles” between a resonant peak and the suppressed background, yielding a reflection difference approaching unity. Although the mathematical form of the TMOKE formula can generate sharp spectral “spikes” when the denominator approaches zero (discussed in Supporting Information S3), we emphasize that the peaks reported here originate from large absolute reflectivity differences, ensuring high signal‐to‐noise ratios for experimental detection.

The angular robustness of the effect is further quantified in Figure 3h. For s‐polarization, the TMOKE peaks consistently exceed 1.7 and remain stable even at large angles up to 40°. This exceptional performance arises from the interplay between mode‐induced near‐field asymmetry and the high‐Q resonant shift. Interestingly, although TMOKE strictly vanishes at normal incidence due to the restoration of spatial inversion symmetry, a significant and measurable signal emerges even at minute angles (e.g., 0.5°). This rapid onset of the MO response occurs because any finite in‐plane wavevector component *k*_*x*_ ≠ 0 is sufficient to break the symmetry and activate the “indirect coupling” mechanism. From a practical perspective, this characteristic is highly advantageous; even with the inherent angular divergence of a Gaussian beam or slight misalignments in an experimental setup, the structure consistently generates detectable and robust TMOKE signals without the need for stringent beam collimation.

To further demonstrate the versatility of the proposed quasi‐2D MO grating, we investigate its performance under p‐polarized incidence. Although the core innovation of this work lies in activating the “forbidden” s‐polarized channel, achieving high‐performance operation in both polarization states is highly desirable for polarization‐insensitive photonic applications.

It is worth noting that our collaborative RCWA‐PSO framework was specifically configured to optimize a broadband low‐reflection background for both s‐ and p‐polarizations simultaneously. Figure 4a displays the reflection map for the foundational 1D MO grating under p‐polarized incidence. The results reveal that this optimized 1D structure provides a broadband and wide‐angle low‐reflectance region, where the reflectance is below 5% across a 300 nm bandwidth (from 1350 to 1650 nm) for incidence angles ranging from −40° to +40°. The resonant modes responsible for this p‐polarized enhancement are analyzed in Figure 4b. The normalized electric field components *|E*_*x*_ */E*_*0*_ *|* and *|E*_*z*_ */E*_*0*_ *|* for the TE‐p and TM‐p modes clearly distinguish their characteristic profiles: the TE‐p mode is dominated by the in‐plane *E*_*x*_ component, whereas the TM‐p mode is characterized by the prevailing out‐of‐plane *E*_*z*_ component. This dual‐polarization “dark background” serves as a universal foundation, ensuring that the MO‐induced frequency shifts in either polarization state lead to near‐limit TMOKE responses.

**FIGURE 4:**
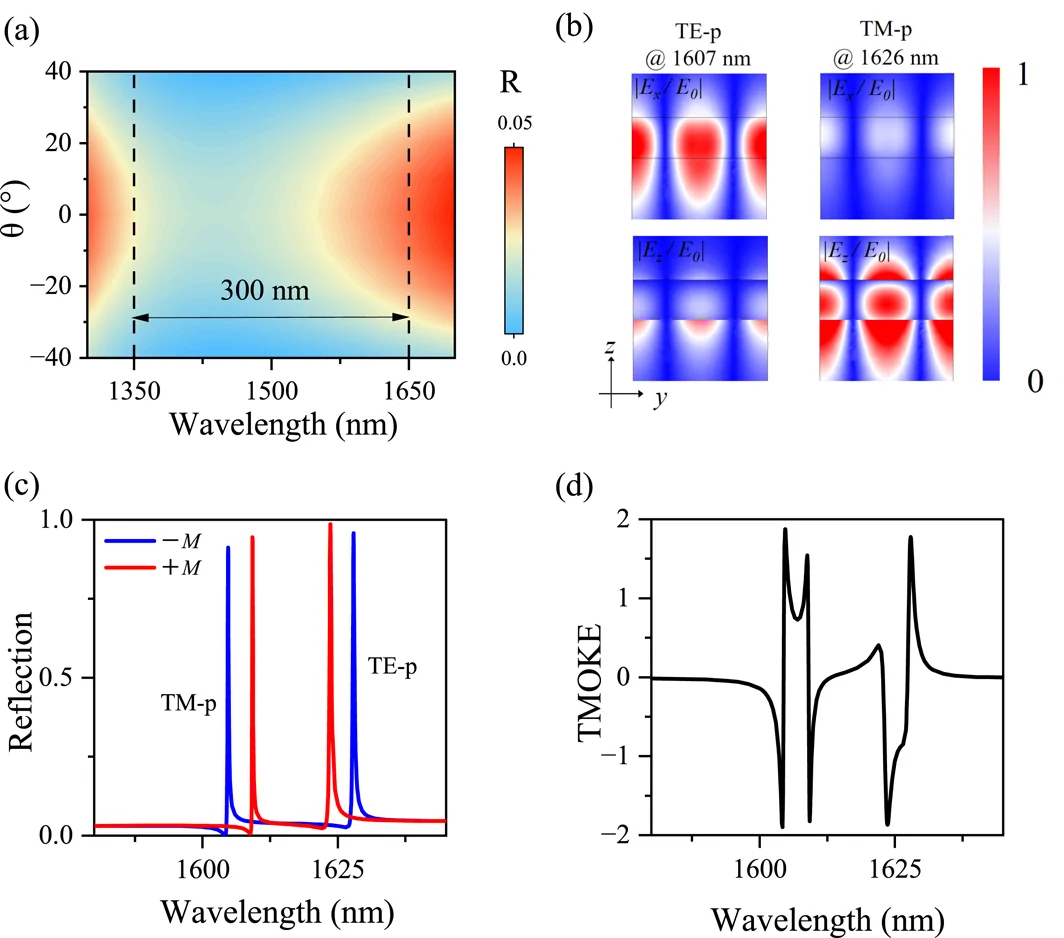
TMOKE enhancement under p‐polarized incidence. (a) Reflection map of the foundational 1D MO grating under p‐polarized incidence as a function of wavelength and incidence angle. (b) Normalized electric field magnitudes *|E*_*x*_ */E*_*0*_ *|* and *|E*_*z*_ */E*_*0*_ *|* of the resonant modes (TE‐p and TM‐p) within the MO layer. (c) Reflection spectra for p‐polarization under reversed magnetization at *θ* = 20°. (d) Corresponding p‐polarized TMOKE spectrum, showing values approaching the theoretical limit of 2.

Figure 4c displays the reflection spectra under reversed magnetization at *θ* = 20° for p‐polarization. Similar to the s‐polarized case, the p‐polarized response also exhibits giant spectral splitting superimposed on a suppressed background. As shown in Figure 4d, the peak TMOKE values for p‐polarization approach the theoretical limit of 2. A TMOKE magnitude exceeding 1 indicates that the absolute reflectance difference is greater than the average reflectance, signifying an exceptionally high modulation depth. This robust performance across a broad angular range enables high‐performance operation without the need for stringent alignment. The p‐polarized reflectance spectrum of the grating and the implementation of its TMOKE tuning are fully documented in the Supporting Information S4.

In practical applications, MO materials such as BIG inevitably exhibit intrinsic optical absorption. This introduces an imaginary part (*ε*_*i*_) to the dielectric constant, which leads to the broadening of resonant modes and a potential reduction in the MO response. To evaluate the experimental relevance and reliability of our design, we performed a systematic parametric study by varying *ε*_*i*_ from 0.001 to 0.01 to examine the robustness of the TMOKE enhancement for both s‐ and p‐polarizations.

Figure 5a presents the reflection spectra under s‐polarized excitation for three graded *ε*_*i*_ values (0.001, 0.005, and 0.01). The results indicate that increasing *ε*_*i*_ leads to a broadening of the resonance and a reduction in the quality factor (Q) from approximately 4000 to 1000. Crucially, even at the highest loss level (*ε*_*i*_ = 0.01), the resonance linewidth remains substantially narrower than the magnetic‐field‐induced spectral splitting. This ensures that the +*M* and −*M* states remain spectrally well‐resolved. As shown in Figure 5b, the peak TMOKE for s‐polarization remains at a giant level across all investigated *ε*_*i*_ values, maintaining over 95% of the ideal lossless performance at *ε*_*i*_ = 0.001. A similar trend is observed for p‐polarized incidence, as illustrated in Figure 5c and 5d. The quality factor decreases from 3000 to 900 with increasing absorption, yet the ± *M* spectra remain clearly separated in the frequency domain. The key to this remarkable robustness lies in our “near‐zero reflection background” strategy.

**FIGURE 5:**
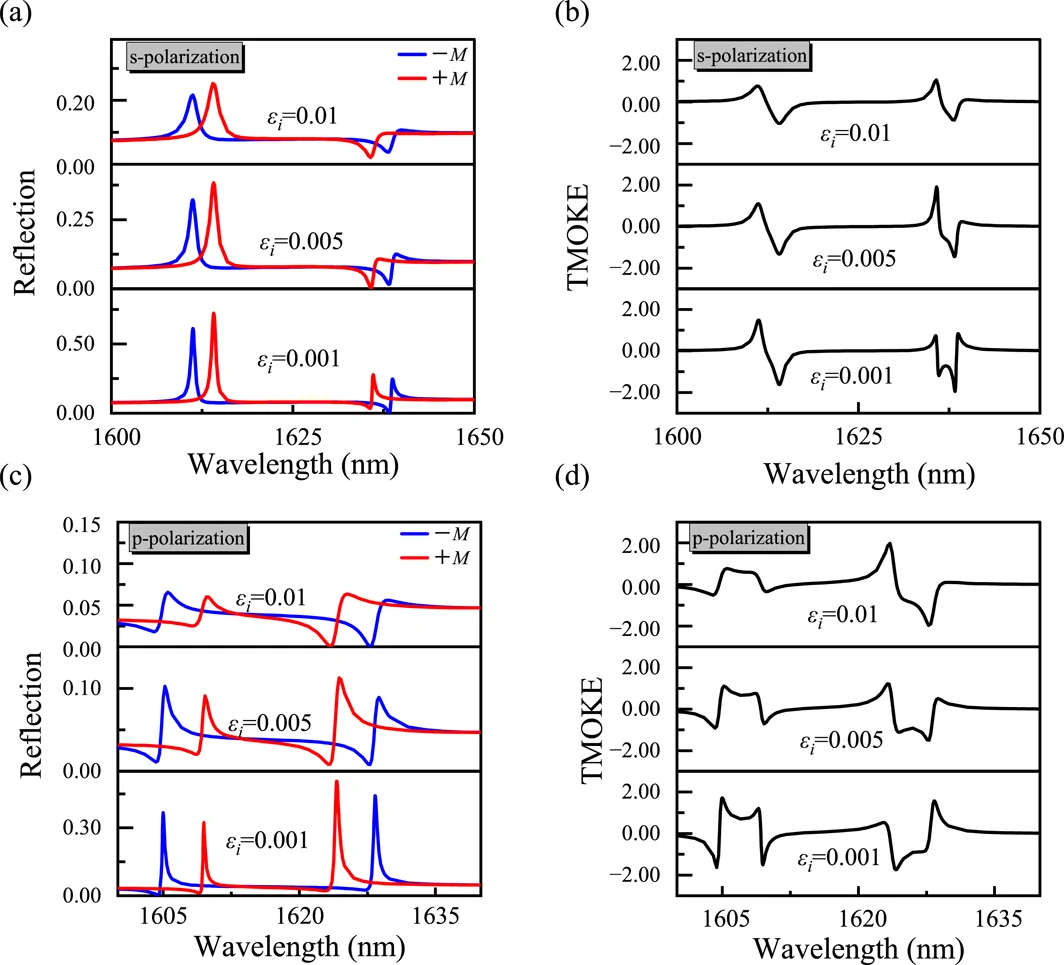
Influence of material intrinsic absorption on the giant TMOKE response at *θ* = 20°. (a) Simulated reflection spectra under s‐polarized incidence for reversed magnetization states (± *M*) and (b) corresponding peak TMOKE values, calculated for varying imaginary parts of the dielectric constant (*ε*_*i*_ = 0.001, 0.005, and 0.01). (c) Simulated reflection spectra and (d) corresponding TMOKE spectra under p‐polarized incidence for the same *ε*_*i*_ values.

These results for both s‐ and p‐polarizations demonstrate that the structure provides a practical and resilient platform for MO enhancement. The consistency in behavior between the two polarization states highlights the universality of our mode‐regulation mechanism. This analysis provides strong “theoretical confidence” that the proposed grating can approach the theoretical performance limit using commonly available, lossy magneto‐optical garnets, without the need for idealistic lossless materials.

Finally, to ensure the practical feasibility of our design, we systematically evaluate its sensitivity to fabrication‐induced structural deviations. We examine five critical parameters: groove length (*l*), groove width (*w*), relative groove position, grating height (*h*), and grating width (*d*). As illustrated in Figure 6a, each parameter is assigned a random deviation relative to its nominal design value. These deviations—defined within a range of ± 20% for groove dimensions, ± 40 nm for relative positioning, and ± 20 nm for the grating profile—are specifically chosen to encompass the typical manufacturing tolerances of electron‐beam lithography (EBL) and dry etching processes. For each parameter, multiple sets of random deviations were generated and incorporated into supercell simulations (comprising three adjacent unit cells) to ensure statistical relevance. The specific values for these random perturbations are detailed in the Supporting Information S5. Figure 6b,c summarize the peak |TMOKE| values extracted for s‐ and p‐polarizations, respectively.

**FIGURE 6:**
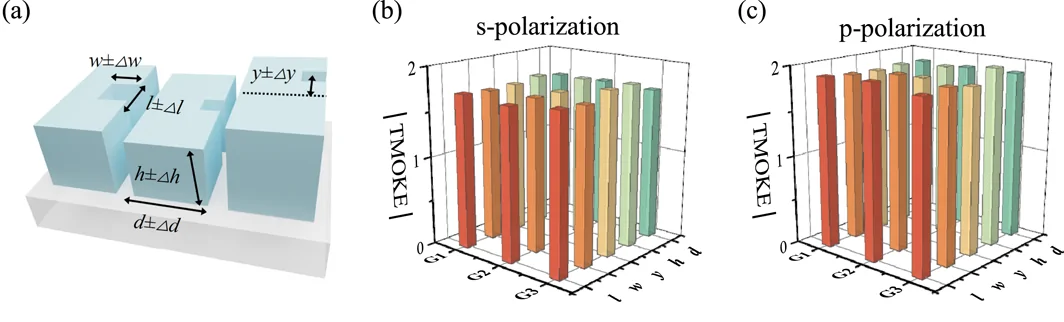
Analysis of various manufacturing defect scenarios that may occur during electron beam lithography. (a) Schematic diagrams of five size deviation models. (b) Values of TMOKE under s‐polarization. (c) Values of TMOKE under p‐polarization.

Remarkably, even in the presence of significant structural imperfections, the TMOKE response remains consistently high (exceeding 1.7) across all simulation batches. This exceptional robustness is a direct consequence of our functional decoupling strategy: because the low‐reflection background and the resonant modes are established by different structural features, small random deviations in the 2D grooves primarily affect the Q‐factor rather than shifting the system away from its near‐zero reflection baseline. Consequently, the giant TMOKE signal is preserved, confirming that our proposed scheme is highly resilient to real‐world manufacturing deviations and provides a robust blueprint for practical experimental realization. The experimental feasibility of such complex nanostructures is well‐supported by recent advancements in nanofabrication. For instance, the nanopatterning of bismuth‐substituted iron garnet (BIG) materials has been successfully demonstrated [25], and quasi‐2D dielectric gratings with similar structural geometries have been realized using standard electron‐beam lithography (EBL) and dry etching processes [32]. These experimental precedents, combined with our rigorous tolerance analysis, confirm that our design provides a robust and reliable blueprint for practical realization.

## 4 Conclusion

In summary, we have demonstrated that the s‐polarized TMOKE can be elevated to its theoretical limit (∼2) through a mode‐driven “indirect magneto‐optical coupling” mechanism. Unlike conventional direct‐coupling schemes, our strategy exploits the spatially asymmetric near‐fields of resonant modes to bypass symmetry‐imposed constraints. By coordinating a foundational 1D grating with periodic grooves etched into the ridges, we achieve a functional synergy: the 1D structure provides a broadband near‐zero reflection background, whereas the periodic grooves enable high‐Q modes propagating along the ridges to induce maximal field asymmetry. Quantitative analysis using a field asymmetry factor confirms that this localized imbalance prevents the spatial cancellation of MO contributions, enabling a giant response. Furthermore, the design is polarization‐insensitive and exhibits exceptional robustness against material losses and fabrication deviations. Given its high performance and demonstrated compatibility with current nanofabrication techniques, this structure opens new avenues for high‐sensitivity MO sensing and integrated nonreciprocal nanophotonics.

## Author Contributions

**Zicheng Yang:** conceptualization, investigation, methodology, validation, software. **Shuqiao Xu:** conceptualization. **Haoshuang Zhong:** software. **Guoyong Zhang:** software. **Hao Chen:** software. **Peng Zhan:** writing – review and editing. **Shuming Wang:** writing – review and editing. **Zhuo Chen:** writing – review and editing. **Zhenlin Wang:** writing – review and editing.

## Funding

This work was supported by the National Key Research and Development Program of China (2022YFA1404300) and the National Natural Science Foundation of China (12174189).

## Conflicts of Interest

The authors declare no conflicts of interest.

## Supporting information


Supporting Information S1

## Data Availability

The data that support the findings of this study are available from the corresponding author upon reasonable request.
