# Supplementary material for: Giant Enhancement of the Anomalous Transverse Magneto‐Optical Kerr Effect in a Magneto‐Optical Grating
Source: Nanophotonics. 2026 May 14;15(10):e70139. doi: 10.1002/nap2.70139 (PMC13642120; doi:10.1002/nap2.70139)
Supplement: Supplementary file 1 [file NAP2-15-e70139-s001.docx]

**1.The theoretical derivation of the coupling between s-polarized light and the magneto-optical medium.**

In order to clarify the TMOKE in metamaterials, we provide a detailed theoretical analysis starting from the Maxwell’s equations. We consider the incident plane perpendicular to the external magnetic field, i.e., the Voigt geometry. This is also the geometric formula for measuring the TMOKE. The permittivity and permeability tensors of magneto-optical materials are:

$$\varepsilon=\left(\begin{matrix} \varepsilon& 0 & -j\gamma\\ 0 & \varepsilon_{y} & 0 \\ j\gamma& 0 & \varepsilon\end{matrix}\right),\mu=\left(\begin{matrix} \mu& 0 & -j\kappa\\ 0 & \mu_{y} & 0 \\ j\kappa& 0 & \mu\end{matrix}\right)$$

where ε and μ are the diagonal components of the permittivity and permeability, γ and κ are the off-diagonal components of permittivity and permeability. The electric and magnetic field vectors can be expressed as $\vec{E}=E_{0}e^{-j\vec{k}\cdot\vec{r}},\vec{H}=H_{0}e^{-j\vec{k}\cdot\vec{r}}$.

Therefore, the Maxwell’s equations can be written as

$$\begin{matrix} \vec{k}\times\vec{E}=k_{0}\vec{\mu}\vec{H} \\ \vec{k}\times\vec{H}=-k_{0}\vec{\varepsilon}\vec{E} \end{matrix}$$

Where k0=ω/c is the free space wave vector, $\vec{k}=\left[k\sin\theta,0,-k\cos\theta\right]^{T}$ is the wave vector.

Then Maxwell’s equations can be factorized into two equations for s and p polarizations:

$\begin{matrix} -k_{z}E_{y}=k_{0}(\mu H_{x}-j\kappa H_{z}) \\ k_{x}E_{y}=k_{0}(\mu H_{z}+j\kappa H_{x}) \\ k_{z}H_{x}-k_{x}H_{z}=-k_{0}\varepsilon_{y}E_{y} \end{matrix}$, $\left(\begin{matrix} -k_{z} & -k_{0}\mu& jk_{0}\kappa\\ k_{x} & -jk_{0}\kappa& -k_{0}\mu\\ k_{0}\varepsilon_{y} & k_{z} & -k_{x} \end{matrix}\right)\left(\begin{matrix} E_{y} \\ H_{x} \\ H_{z} \end{matrix}\right)=0$ (1.1) s-polarization
$\begin{matrix} k_{z}H_{y}=k_{0}(\varepsilon E_{x}-j\gamma E_{z}) \\ k_{x}H_{y}=-k_{0}(\varepsilon E_{z}+j\gamma E_{z}) \\ k_{z}E_{x}-k_{x}E_{z}=k_{0}\mu_{y}H_{y} \end{matrix}$ , $\left(\begin{matrix} -k_{0}\mu_{y} & k_{z} & -k_{x} \\ -k_{z} & k_{0}\varepsilon& -jk_{0}\gamma\\ k_{x} & jk_{0}\gamma& k_{0}\varepsilon\end{matrix}\right)\left(\begin{matrix} H_{y} \\ E_{x} \\ E_{z} \end{matrix}\right)=0$ (1.2) p-polarization

From equations (1.1), we see only the μ, κ, and ε components are present, while γ is absent from the equations for s-polarization. Whereas only the μ, ε and γ components are present, while κ is absent from the equations for p-polarization. Given the homogeneous equation, they will have non-trivial solutions when the determinant of the coefficients vanishes. Therefore, when the incident light is s-polarized, the magnetization vector is strictly parallel to the electric field component of the light. In the optical frequency range, the magnetic permeability of traditional materials is always 1. It is impossible to excite effective coupling of the off‑diagonal dielectric tensor elements. As a result, anomalous TMOKE has long been absent under s‑polarization. All the rich electric field components in the guided mode and their controllable symmetries provide a feasible physical path for compensating the absence of specific field components.

**2. Effect of high-reflectivity** **grating background on TMOKE enhancement.**

Fig.S2. Reflection spectra and TMOKE signals (under s-polarized incidence) in grating background.

We designed a magneto-optical grating with a reflectivity of approximately 60% and observed that both the TMOKE enhancement and the differential reflection signal of the excited mode decreased significantly. The grating has a period of 800 nm, a height of 900 nm, and a duty cycle of 0.67. The material parameters remain the same as those in the main text.

**3. Reflection and TMOKE spectra at an incident angle of 20° under s-polarization.**

Fig.S3. S-polarized reflection and TMOKE spectra at an incident angle of 20°. (a) Reflection and TMOKE spectra recorded under positive and negative magnetic fields. The blue shaded region highlights the spectral range where multiple TMOKE peaks emerge. (b) Magnified view of the highlighted region.

**4. TMOKE enhancement under p-polarized excitation.**

Fig.S4. (a) Reflectivity of the MO-grating under p-polarized incidence as a function of incident angle; (b) Reflection spectra versus wavelength for varying *p_y_* thicknesses at a fixed incident angle of 20° under p-polarized incidence.

**5. Specific numerical settings for deviations of five structural parameters.**

TABLE 1. Specific numerical settings for deviations of five structural parameters.

|  |  | *l* (nm) | *w* (nm) | *y* (nm) | *h* (nm) | *d* (nm) |
| --- | --- | --- | --- | --- | --- | --- |
| G1 | 1 | 90 | 86 | -34 | -18 | -20 |
|  | 2 | 98 | 114 | -28 | 15 | 14 |
|  | 3 | 108 | 120 | -14 | 4 | 5 |
| G2 | 1 | 88 | 97 | 40 | 12 | 5 |
|  | 2 | 118 | 111 | -6 | -13 | -8 |
|  | 3 | 106 | 108 | -12 | 17 | 13 |
| G3 | 1 | 104 | 115 | -28 | 3 | 18 |
|  | 2 | 84 | 82 | 15 | -8 | -7 |
|  | 3 | 110 | 102 | 40 | 20 | -6 |
